# A Universal Formulary: Containing the Methods of Preparing and Administering Officinal and Other Medicines

**Published:** 1850-09

**Authors:** 


*A Universal Formulary: Containing the methods of preparing and administering officinal and other medicines. The whole adapted to Physicians and Pharmaceutists.* By R. Eglesfeld Griffith, M. D., Philadelphia. Lea & Blanchard, 1850. 8 vo., pp. 567.

"The design of this work (to quote the Author's words) is to present a compendious collection of formula, and pharmeceutic processes, with such additional information as may render it useful to the physician and apothecary; and the principal aim has been to select materials most generally applicable, and of practical utility."

In the introduction are presented tables and observations on the weights and measures employed for pharmaceutical purposes in the United States, and in foreign countries; and, also, a copious vocabulary of abbreviations and Latin terms employed in writing prescriptions. The latter are now but little used by practitioners, and, in our opinion, the sooner they fall into complete disuse the better. Still, as they enter more or less into medical literature, is is convenient and important that they should be known, and hence it is desirable to be able to refer to a vocabulary occasionally to refresh the memory. The author has added to the introduction a few observations on the management of the sick-room, and on the administration of remedies, which are judicious and appropriate.

The Formulary occupies about one half the volume. It is arranged alphabetically according to the pharmaceutic names adopted in the United States Pharmacopæia. In each formula the English names for the articles are used, and the quantities are expressed in words, instead of the usual signs. The author thinks that this change, which has been made in France, should be adopted by other countries, believing that it would tend to obviate mistakes in writing prescriptions. We are disposed to concur in this opinion, and can see no occasion for regrets, if the " abbreviated cabalistic terms" now used, were to be discarded.

Following the Formulary are a list of incompatibles; a posological table, or title of doses; a table of pharmaceutical names which differ in the United States, and the London, Edinburgh, aud Dublin Pharmacopæies; officinal preparations and directions; poisons; index of diseases and their remedies; index of pharmaceutical and botanical names, and a copious general index.

From the foregoing statement of contents, the reader will perceive that the work is calculated, in several points of view, to prove convenient and useful to the medical practitioner. And, from its intrinsic value, the work derives an interest from the fact that it was the last effort of the author to render useful service to the Profession of which he was an industrious and distinguished member, his decease being almost coincident with its publication. Dr. Griffith made several valued contributions to medical literature, among which is a work on medical botany, which is highly esteemed. As remarked by a cotemporary, he was emphatically a working man, and his loss is much to be deplored.

